# Investigation of the *Fusarium virguliforme* Transcriptomes Induced during Infection of Soybean Roots Suggests that Enzymes with Hydrolytic Activities Could Play a Major Role in Root Necrosis

**DOI:** 10.1371/journal.pone.0169963

**Published:** 2017-01-17

**Authors:** Binod B. Sahu, Jordan L. Baumbach, Prashant Singh, Subodh K. Srivastava, Xiaoping Yi, Madan K. Bhattacharyya

**Affiliations:** 1 Department of Agronomy, Iowa State University, Ames, Iowa, United States of America; 2 Interdepartmental Genetic Program, Iowa State University, Ames, Iowa, United States of America; College of Agricultural Sciences, UNITED STATES

## Abstract

Sudden death syndrome (SDS) is caused by the fungal pathogen, *Fusarium virguliforme*, and is a major threat to soybean production in North America. There are two major components of this disease: (i) root necrosis and (ii) foliar SDS. Root symptoms consist of root necrosis with vascular discoloration. Foliar SDS is characterized by interveinal chlorosis and leaf necrosis, and in severe cases by flower and pod abscission. A major toxin involved in initiating foliar SDS has been identified. Nothing is known about how root necrosis develops. In order to unravel the mechanisms used by the pathogen to cause root necrosis, the transcriptome of the pathogen in infected soybean root tissues of a susceptible cultivar, ‘Essex’, was investigated. The transcriptomes of the germinating conidia and mycelia were also examined. Of the 14,845 predicted *F*. *virguliforme* genes, we observed that 12,017 (81%) were expressed in germinating conidia and 12,208 (82%) in mycelia and 10,626 (72%) in infected soybean roots. Of the 10,626 genes induced in infected roots, 224 were transcribed only following infection. Expression of several infection-induced genes encoding enzymes with oxidation-reduction properties suggests that degradation of antimicrobial compounds such as the phytoalexin, glyceollin, could be important in early stages of the root tissue infection. Enzymes with hydrolytic and catalytic activities could play an important role in establishing the necrotrophic phase. The expression of a large number of genes encoding enzymes with catalytic and hydrolytic activities during the late infection stages suggests that cell wall degradation could be involved in root necrosis and the establishment of the necrotrophic phase in this pathogen.

## Introduction

Sudden death syndrome (SDS) is a serious, emerging soybean disease. It is caused by *Fusarium virguliforme*, which prefers cold and wet climates for developing the disease [1]. In the United States, the disease was first reported in Arkansas in 1971 [2]. Subsequently, the disease spread northward and now it has been reported in soybean growing areas in the United States and Canada [2–4]. In 2010, the estimated soybean yield suppression from SDS was estimated at 2.1% of total yield valued at $0.82 billion dollars [5]. SDS can be divided into two components: (i) root necrosis and (ii) foliar SDS. Root symptoms consist of root necrosis with vascular discoloration spreading to a few nodes and internodes of the stem. At the initial stage, foliar SDS is characterized by interveinal chlorosis and necrosis of leaves, and in severe cases by flower and pod abscission [6].

*F*. *virguliforme* (formerly known as *F*. *solani* f. sp. *glycines*) is a hemibiotrophic fungal pathogen with an initial biotrophic phase [7–10]. Although symptoms appear on soybean leaves, the pathogen has never been isolated from the diseased foliar tissues. The pathogen remains in infected roots and produces toxins that are involved in foliar SDS development [11–14]. *F*. *virguliforme* secretes several proteins, including phytotoxins, into the culture medium [12]. Treatment of cut soybean seedlings with cell-free *F*. *virguliforme* culture filtrates results in degradation of the Rubisco large subunit in the presence of light [15]. A major *F*. *virguliforme* proteinacious toxin, FvTox1, has recently been shown to cause foliar SDS [11]. It is an acidic 13.5 kDa protein, which is secreted to the infected soybean roots and *F*. *virguliforme* culture media [11]. Expression of a single-chain variable fragment (scFv) antibody against this toxin enhanced foliar SDS resistance in transgenic soybean plants [16]. Knockout mutants created by homologous recombination established that FvTox1 is a major virulence factor for foliar SDS development in soybean [17]. How the toxin causes foliar SDS is not yet known and there may be additional toxins that contribute to development of foliar SDS [14].

SDS management options are limited. Genetic resistance to *F*. *virguliforme* is partial and encoded by a large number of quantitative trait loci (QTL), each contributing only a small amount of resistance [18–23]. No single genes conditioning SDS resistance have been identified and most unlikely are available. Since *F*. *virguliforme* is a root pathogen and remains in soil, little can be done once the toxins start to induce foliar SDS.

Significant changes in expression patterns of 2,467 soybean genes were observed during the soybean—*F*. *virguliforme* interaction [24]. In addition to the soybean genes, 93 small RNA and 42 micro RNA with putative target sites in the soybean genome were identified [24]. Toxins of the *F*. *virguliforme* culture filtrates induce defense pathways in leaves [25]. ESTs upregulated during root responses of recombinant inbred lines (RILs) obtained from a cross of ‘Essex' x 'Forrest’ showed the involvement of resistance gene analogs, genes governing signal transduction, plant defense, cell wall synthesis and transport of metabolites [8].

Sequencing of the *F*. *virguliforme* genome revealed 14,845 predicted genes [26]. However, the expression of *F*. *virguliforme* genes during infection in soybean and their possible involvement in infection of soybean roots has not yet been investigated. In this study, deep sequencing of transcripts collected from *F*. *virguliforme* infected root tissues of a highly susceptible soybean cultivar ‘Essex’ was conducted to identify candidate virulence genes involved in root necrosis. Comparison of the transcriptome of *F*. *virguliforme* infected root tissues with that of germinating conidia and mycelia identified 1,886 *F*. *virguliforme* genes that are induced at least two fold during infection. Gene ontology analyses of genes with ≥10-fold increase in expression levels in infected root tissues vs. germinating conidia and mycelia were classified into three major functional categories: cell-wall modification, oxidative stress, and membrane transport. A large number of the infection-induced genes are predicted to have hydrolytic activities and may play a major role in establishing the necrotrophic phase of the pathogenic fungus. The candidate virulence genes identified in this study lay the foundation for identification of *F*. *virguliforme* virulence mechanisms and the molecular basis of the root necrosis caused by this soybean pathogen.

## Materials and Methods

### Plant materials and inoculation

Soybean [*Glycine max* (L.) Merrill] cultivar Essex, highly susceptible to *F*. *virguliforme*, was sown in vermiculite under dark conditions at 22°C. The 7-day old seedlings were uprooted and used for root infection. *F*. *virguliforme* Mont-1 isolate was maintained on Bilay agar plates [(0.1% KH_2_PO_4_ (w/v), 0.1% KNO_3_ (w/v), 0.05% MgSO_4_ (w/v), 0.05% KCl (w/v), 0.02% starch (w/v), 0.02% glucose (w/v), 0.02% sucrose (w/v) and 2% agar(w/v)] and sub-cultured on 1/3 PDA agar plates [0.04% potato starch (w/v), 0.2% glucose (w/v), 2% agar (w/v)]. On 1/3 PDA plates the *F*. *virguliforme* Mont-1 isolate produced the characteristic blue mass containing conidia after two weeks of growth. The roots of 10 etiolated 7 day old Essex seedlings, grown in coarse vermiculites, were either infected with *F*. *virguliforme* Mont-1 conidia suspensions (10^7^ spores/mL) or treated with sterile water for specified periods under dark conditions prior to harvesting for RNA isolation [11]. Harvested roots infected with conidia were thoroughly washed prior to freezing in liquid nitrogen for RNA preparation.

### RNA extraction from germinating conidia, mycelia and root samples

**Fig 1:**
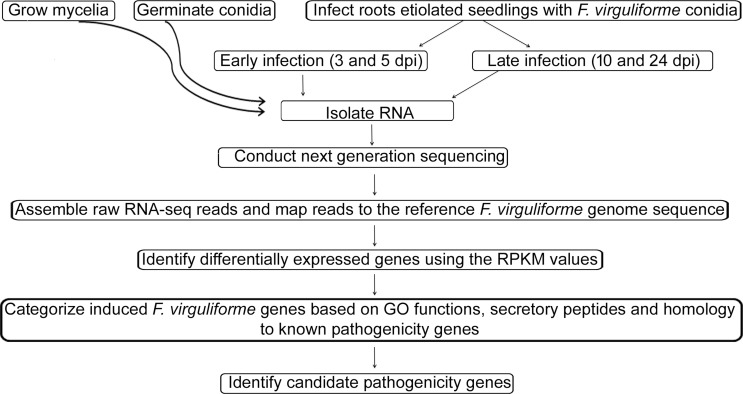
Flow diagram of the steps followed in conducting the transcriptomic analysis. (i) RNA samples were isolated from germinating spore, mycelia, early stage of infection and late stage of infection (details in materials and methods). (ii) RNA samples were reverse transcribed into cDNA and sequenced using Solexa sequencing platform. (iii) Assembling of the RNA sequence datasets and mapping to reference sequence (http://fvgbrowse.agron.iastate.edu/) was conducted using Bowtie. (iv) Determined the expression levels of individual genes (RPKM values). (v) Validation of the RNA sequence data by PCR analyses (iv) Conducted functional categorization of *F*. *virguliforme* genes by BLAST2GO analyses and validated expression levels of a few selected genes from different functional categories. (v) Identification of putative *F*. *virguliforme* virulence genes. dpi, day post inoculation.

*F*. *virguliforme* infected root samples were collected at 3, 5, 10 and 24 days post inoculation (dpi) from three independent experiments. The overview of the experimental strategy is shown in Fig 1. Necrotic symptoms were visible at 10 dpi with gradual spreading of root rot symptoms until 24 dpi. RNA samples of infected roots from three experiments, collected 3 and 5 dpi, were bulked and named as “early infection” whereas the pooled RNA sample isolated from the infected roots, collected 10 and 24 dpi, were termed as “late infection”. Early time points (3 and 5 dpi) were selected to study the putative biotrophic phase with very little root necrosis; while late time points (10 and 24 dpi) were selected for investigating the necrotrophic phase (S1 Fig).

For semi-quantitative RT-PCR and quantitative (qRT-PCR) analyses, root samples were harvested at 1, 3, 5 and 10 dpi either following *F*. *virguliforme* infection or treatment with water. In RT-PCR, we included 1 dpi in RT-PCR to determine the early changes in gene expression while 10 dpi was considered to represent the late time point ignoring 24 dpi treatment. In each experiment, roots of five plants were pooled for each treatment and frozen in liquid nitrogen and stored at -80°C until further use.

Germinating conidia were grown for 12 h in liquid modified Septoria medium (MSM) [13]. Mycelia were harvested from spores grown in MSM liquid media for two weeks. Total RNAs were isolated from the germinating conidia, mycelia and infected root tissues using TRIzol Reagent (Invitrogen, Carlsbad, CA, U.S.A.). The quality of RNA samples was determined by running RNAs on a denaturing agarose gel.

### cDNA library preparation for sequencing transcripts

10 ug total RNAs from early and late time points of *F*. *virguliforme* infected soybean root tissues, germinating conidia, and mycelia were used to purify poly (A)^+^ RNAs using oligo (dT) attached to magnetic beads (Promega, Madison, WI). Poly (A)^+^ RNAs were fragmented into short sequences in the presence of divalent cations at 94°C for 5 min. RNA samples were reverse transcribed using a cDNA synthesis kit from Illumina (Illumina, Inc. San Diego, CA, U.S.A.).

### Sequencing of the *F*. *virguliforme* transcripts isolated from infected soybean roots, germinating conidia and mycelia

cDNAs of an individual RNA sample were sequenced in a single lane of the Illumina NGS platform GAII (Illumina, Inc. San Diego, CA, U.S.A.) at the DNA Facility, Iowa State University. The raw sequence reads were generated using the Solexa GA pipeline 1.6 (deposited in GEO; accession GSE86201). The draft *F*. *virguliforme* genome sequence (available at http://fvgbrowse.agron.iastate.edu/gb2/gbrowse/fvirguli/ [26]) has been shown to contain 14,845 predicted genes [26]. Reads with quality scores more than Phred 33 were used to map transcripts to the cDNA sequences of predicted *F*. *virguliforme* genes using the Bowtie program [27] with the default parameters. The generated SAM (Sequence Alignment/Map) output for each condition was used to extract mapped reads (S1 Table) for corresponding genes using a custom script [28]. The reads per kb per million reads (RPKM) for each gene was calculated according to the formula R = 10^9^C/NL [29], where C is the number of mappable reads aligned onto the exonic sequence of a gene, N is the total number of mappable reads in the sample, and L is the length of the gene. CIMminer was used to generate color-coded Clustered Image Maps (CIMs) ("heat maps") representing "high-dimensional" data sets such as gene expression profiles (http://discover.nci.nih.gov/cimminer/). A heat map was generated in one matrix CIM for the normalized values for each gene after RPKM analysis [30]. TargetP1.1 (http://www.cbs.dtu.dk/services/TargetP/) set for non-plant as organism with no cutoffs, winner-takes-all was used to identify the candidate secreted proteins.

### Identification of the candidate *F*. *virguliforme* virulence genes

We looked for infection-induced *F*. *virguliforme* genes that showed high identity to functionally characterized virulence genes by running the NCBI BlastX program against the non-redundant protein sequences (nr) database (http://blast.ncbi.nlm.nih.gov/Blast.cgi).

### Semi-quantitative RT-PCR and quantitative real-time PCR (qRT-PCR) analyses

Semi-quantitative PCR of several induced *F*. *virguliforme* genes was conducted to validate the expression profiles deciphered from deep sequencing of single RNA samples pooled from three biological experiments. Genes were selected randomly from individual functional categories. Total RNA samples were isolated from the *F*. *virguliforme*-infected or water treated etiolated root samples of soybean cv. Essex at 1, 3, 5 and 10 days post inoculation or water treatment by using TRIzol (Invitrogen, Carlsbad, CA, U.S.A.). *E*. *coli* DNase I treatment was performed in order to remove any contaminating genomic DNA from the total RNA samples (Invitrogen, Carlsbad, CA, U.S.A.). cDNA was prepared from individual RNA samples using random primers (Invitrogen, Carlsbad, CA, U.S.A.). cDNA samples were used to conduct semi-quantitative RT-PCR at 94°C for 2 min, and then 35 cycles of 94°C for 30 s, 50°C or 55°C for 30 s and 72°C for 30 s then 72°C for 10 min. Primers for RT-PCR are listed in S2 Table. The amplified products were resolved on a 2% (w/v) agarose gel through electrophoresis at 8 V/cm. Normalization of the gene expression for *F*. *virguliforme* genes in qRT-PCR was carried out using the *FvTox1 (g6924)* transcript levels because the transcript levels of *FvTox1* do not change during the time-course of infection (S2 Fig). qRT-PCR for a few selected genes were conducted on an iCycler sequence detection system (Bio-Rad; Hercules, CA, U.S.A) using SYBR Green fast qPCR master mix (Bio-RAD; Hercules, CA, U.S.A).

## Results

The goal of this study was to identify candidate pathogenicity genes for understanding the mechanisms used by *F*. *virguliforme* to cause root necrosis in soybean. Seven day-old etiolated seedlings were inoculated with the *F*. *virguliforme* Mont-1 isolate showed symptoms on the 10th day after inoculation (S1 Fig). Infected seedlings showed very mild symptoms until five day post inoculation (dpi). Severe root necrosis was observed by 10 dpi. Therefore, we classified the infected seedlings to two groups: (i) infected roots harvested 3-d and 5-d post inoculation as early infection phase (early infection) and (ii) 10-d and 24-d post inoculation as the late infection phase (late infection) (Fig 1; S1 Fig). We consider that during the early infection phase the pathogen is most likely in the biotrophic phase and turns to the necrotrophic phase at least by 10-d post inoculation. In order to identify most of the *F*. *virguliforme* genes induced during the infection process, deep sequencing of individual RNA samples was conducted. Since only a small proportion of the RNA transcripts of infected soybean root tissues is encoded by *F*. *virguliforme* genes, deep sequencing of an individual RNA sample collected from three biological replications was conducted in a single lane to detect transcripts of most transcribed genes including those that are expressed at low levels. Since the deep-transcript sequencing was conducted only once, we could not conduct any statistical analysis. We considered only those genes as expressed genes that have shown to contain at least three sequence reads. We validated the transcriptomic data by conducting semi-quantitative RT-PCR and qRT-PCR analyses of 34 and eight *F*. *virguliforme* genes, respectively. Furthermore, only *F*. *virguliforme* genes showing at least 10-fold or more changes in transcript levels between infected roots and average expression levels of germinating conidia and mycelia were considered as differentially expressed genes (DEGs) for GO term enrichment analyses.

### *F*. *virguliforme* gene expression patterns among germinating conidia, mycelia and infected soybean roots

**Fig 2:**
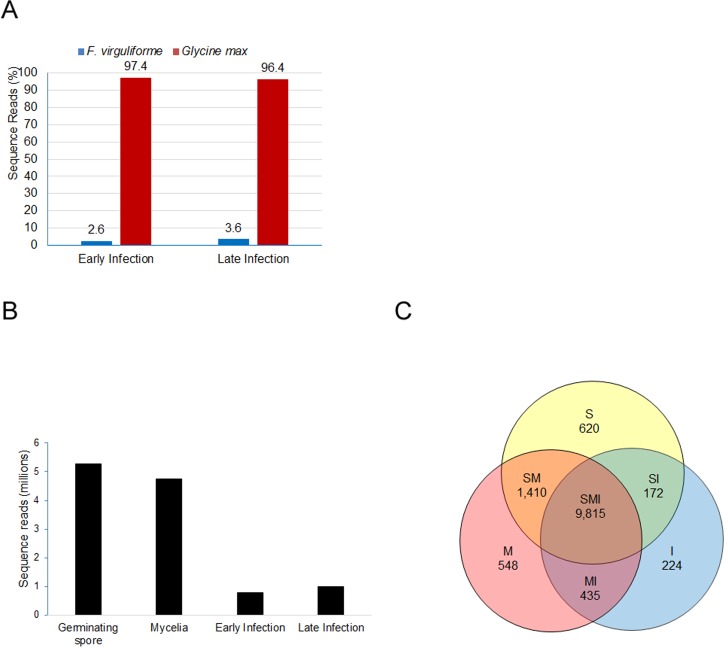
Transcript profiles of the *F*. *virguliforme* genes in various tissues. (A). Percentage sequence reads of *F*. *virguliforme* and soybean genes during infection. (B). Sequence reads of *F*. *virguliforme* genes among various tissue samples. (C). Venn diagram showing unique and common *F*. *virguliforme* genes among germinating spore, mycelia and infected soybean roots. S, genes expressed only in germinating conidia; M, genes expressed only in mycelia; I, genes expressed in infected roots; SM, genes expressed in both spores and mycelia; SI, genes expressed in both spores and infected roots; MI, genes expressed in mycelia and infected roots; SMI, genes expressed in spores, mycelia and infected roots. The genes with a minimum of three reads were considered in developing the Venn diagram. The 984 genes having sequence reads below three were not considered for the Venn diagram.

The numbers of *F*. *virguliforme* and soybean sequence reads were calculated for early and late infection stages. *F*. *virguligforme* transcripts comprised less than 4% of the total transcripts in infected soybean roots (Fig 2A). An increased proportion of *F*. *virguliforme* transcripts was detected in late infection as compared to the early infection stage (Fig 2A). The sequence reads of four tissue samples: (i) germinating spores, (ii) mycelia; (iii) early infection, and (iv) late infection are presented in Fig 2B. Of the 14,845 predicted genes, 13,224 *F*. *virguliforme* genes were expressed among the three-tissue samples. Of the 13,224 *F*. *virguliforme* expressed genes, 9,815 genes were expressed in all samples. Eighty-one percent of *F*. *virguliforme* (12,017 genes) were expressed in germinating spores (Table 1). Among these genes, 620 genes were specifically expressed in germinating conidia (Table 1; Fig 2C). In mycelia, 82% of the predicted *F*. *virguliforme* genes (12,208 genes) were expressed, of which 548 (Fig 2C) are unique to mycelia (Table 1; Fig 2C). Of the 14,845 predicted *F*. *virguliforme* genes, 10,626 (72%) were expressed in infected tissues, of which 224 were expressed only in infected roots. The number of expressed genes was increased during infection from 64% of the predicted *F*. *virguliforme* genes during early infection to 70% in the late infection stage (Table 1). Among the 14,845 predicted *F*. *virguliforme* genes, 2,578 were expressed only in germinating spores and mycelia and are most likely involved in fungal growth and development (Fig 2C).

**Table 1:** *F*. *virguliforme* genes expressed in germinating conidia, mycelia and infected soybean root tissues.

Sample	Number of genes	% of genes expressed	Number of unique genes
Germinating conidia	12,017	81	620
Mycelia	12,208	82	548
Infection	10,626	72	224
(a) Early infection	9,545	64	18
(b) Late infection	10,344	70	79

### Identification of differentially expressed *F*. *virguliforme* genes during infection

To quantify the expression patterns of *F*. *virguliforme* genes, a digital measure of relative gene expression was applied [31]. Typically, large genes have more reads than small genes even if they have the same transcript levels. To avoid this gene size-associated read number bias, the RPKM value for each gene was calculated as described in materials and methods. Fold changes in gene expression during early and late infection stages was calculated by comparing RPKM values of individual genes in infected roots with the average RPKM values of the corresponding genes in spores and mycelia. Of the 10,626 genes expressed in infected roots, 1,886 genes showed at least a 2-fold increase in expression levels when compared to germinating conidia and mycelia (Table 2). Of these 1,886 genes, 80 showed over 50-fold, and 33 showed over 100-fold induction in infected roots as compared to their corresponding average transcript levels in germinating conidia and mycelia (Table 2). In infected soybean roots, expression of 4,204 genes, transcribed in germinating spores and mycelia, were suppressed during infection (Table 2).

**Table 2:** Up or down regulated *F*. *virguliforme* genes during infection compared to germinating conidia and mycelia.

Fold	Number of genes up regulated	Number of genes down regulated
2	1,886	4,204
5	665	1,062
10	369	490
50	80	123
100	33	79
250	8	44
>500	0	20

### Semi quantitative RT-PCR and qRT-PCR validation

**Fig 3:**
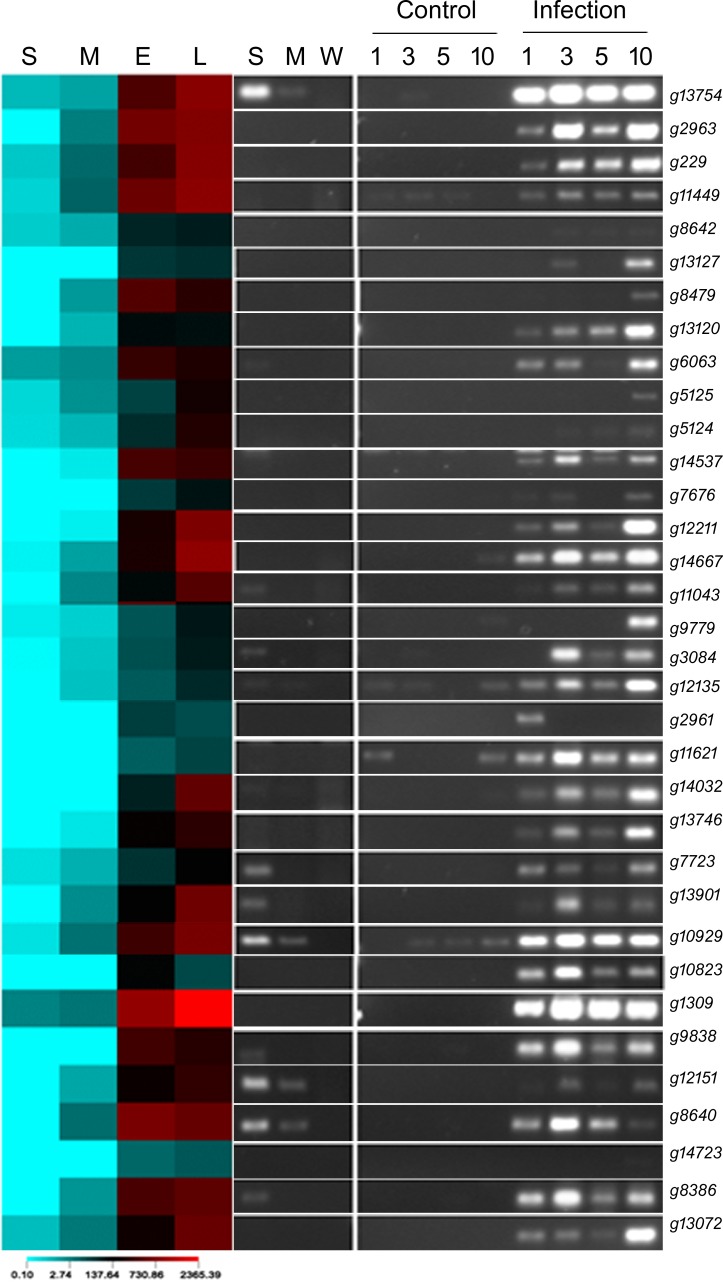
Semi-quantitative RT-PCR of selected genes from various functional categories of genes. On the left, heat map of transcript levels of 34 randomly selected genes in RPKM values is shown. A few genes with unknown functions were also included. On the right, semi-quantitative RT-PCR data of the selected genes are presented. S; germinating conidia, M; mycelia, E; early stage of infection, L; late stage of infection, W; negative control; Control, roots of etiolated soybean (Essex) seedlings were treated with water for 1, 3, 5, and 10 days prior to harvesting; Infection, roots of etiolated soybean (Essex) seedlings were infected with *F*. *virguliforme* (10^7^ spores/mL) for 1, 3, 5, and 10 days prior to harvesting.

To validate the expression data gathered by deep-sequencing of transcripts, 34 genes with ≥10-fold average induction in infected roots as compared to the corresponding averages in germinating spores and mycelia were selected to conduct semi-quantitative RT-PCR (S2 Table). Expression patterns revealed by RT-PCR analyses were consistent with the digital expression data of 30 of the 34 selected genes (Fig 3). Four failed to show expression in RT-PCR, which could be due to primer- or PCR-condition-related issues. For 11 *F*. *virguliforme* genes, we observed amplification of transcripts collected from germinating spores in RT-PCR. The RPKM values of these 11 genes were very low in germinating spores (Fig 3).

**Fig 4:**
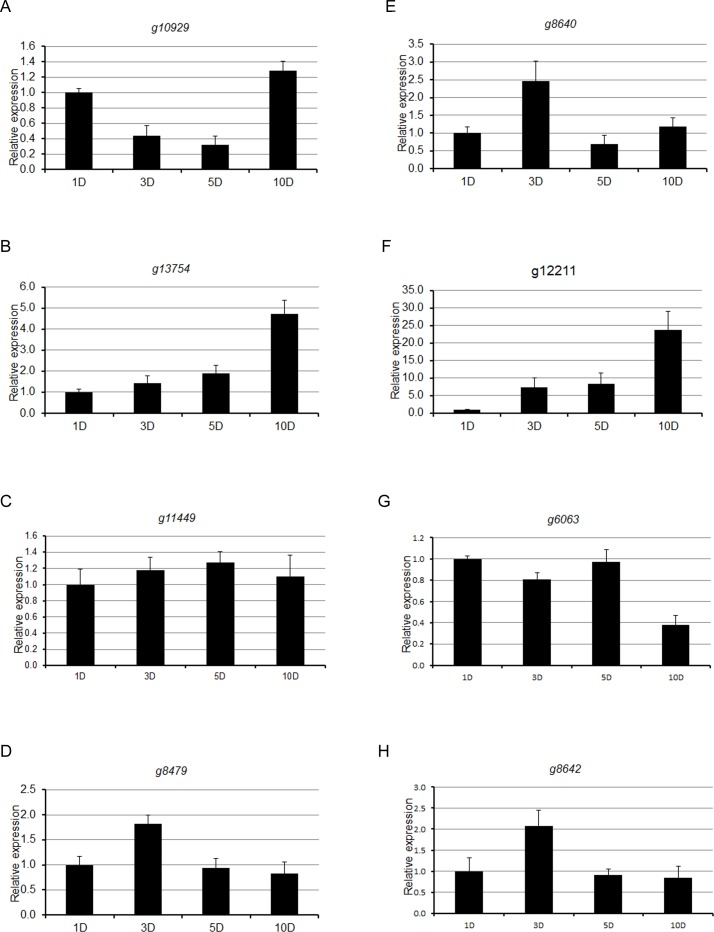
Quantitative RT-PCR of a few selected genes, evaluated in RT-PCR analyses. Quantitative RT-PCR (qRT-PCR) was performed for a few selected virulence genes using gene specific primers to validate semi-quantitative RT-PCR data. (A) *g10929*, (B) *g13754*, (C) *g11449*, (D) *g8479*, (E) *g8640*, (F) *g12211*, (G) *g6063* and (H) *g8642*. The *Fusarium GAPDH* gene was used as an internal control. Relative gene expression levels were determined in proportions to levels of corresponding levels of 1 d *F*. *virguliforme-*infected samples, which are defined as 1. Error bars represent standard deviation (SD) of two independent biological replicates (n = 6).

To validate the semi-quantitative expression data gathered by RT-PCR, qRT-PCR was conducted on a few randomly selected expressed genes (Fig 4), RT-PCR data of which are shown in Fig 3. Overall, expression pattern of *F*. *virguliforme* genes determined by RT-PCR was in agreement with the expression pattern of the selected genes in qRT-PCR analysis. Our RT-PCR and qPCR data validated the transcriptomic data gathered from single RNA samples, pooled from three biological replications (Figs 3 and 4).

### Blast2GO Functional categorization of induced *F*. *virguliforme* genes

**Fig 5:**
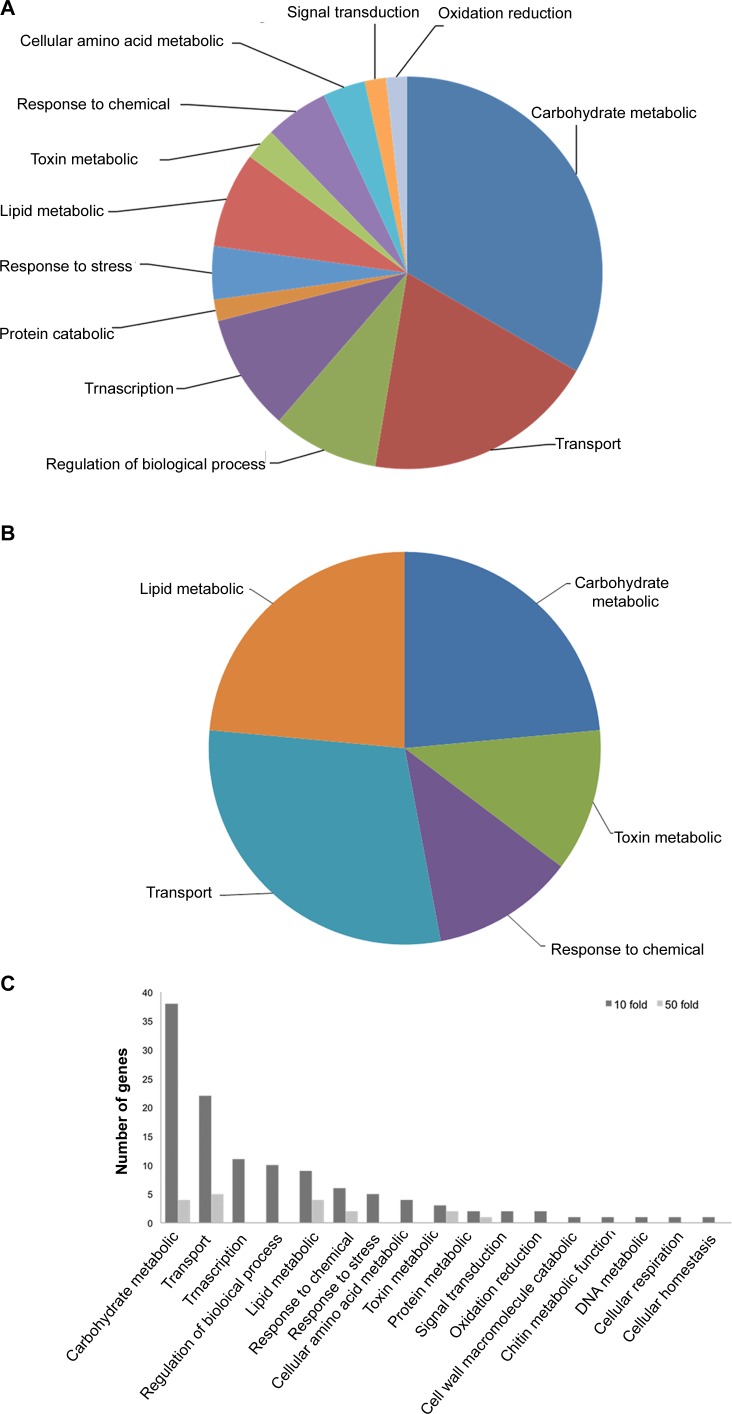
Categories of *F*. *virguliforme* genes based on BLAST2GO analyses for biological processes. A, Categories of *F*. *virguliforme* genes with 10-fold induction. B, Categories of *F*. *virguliforme* genes with 50-fold induction. C, Percentage of genes distributed under different functional categories for biological process are presented as bar diagram for *F*. *virguliforme* genes with 10 and 50 fold induction over mycelia and germinating conidia.

Gene ontology associates molecular functions, cellular components and biological processes to a gene if it shows high sequence identity to previously characterized genes. We used Blast2GO to classify the *F*. *virguliforme* infection-induced genes based on their biological processes and molecular function [32]. *F*. *virguliforme* genes with ≥10 and ≥50 fold increase in expression in infected roots as compared to germinating conidia and mycelia were classified for biological processes and molecular functions to understand the overall basic mechanisms of virulence for this fungal pathogen. Considering the availability of transcript sequences for only a single pooled sample of RNAs, genes with lower than 10 fold changes in expression were not considered in this study. Among the 369 genes with ≥10-fold up-regulation, 273 genes (79%) could be classified by Blast2GO analysis into 17 biological process groups. The most prominent categories of genes are involved in: (i) carbohydrate metabolic processes, (ii) transport, (iii) transcription, (iv) regulation of biological processes, and (v) lipid metabolic processes (Fig 5A and 5C).

Of the 80 genes with ≥50-fold induction, 56 genes (79%) could be classified. These genes were classified into five GO categories: (i) carbohydrate metabolism, (ii) transport, (iii) lipid metabolism processes, (iv) toxin metabolism, and (v) response to chemicals (Fig 5B and 5C).

**Fig 6:**
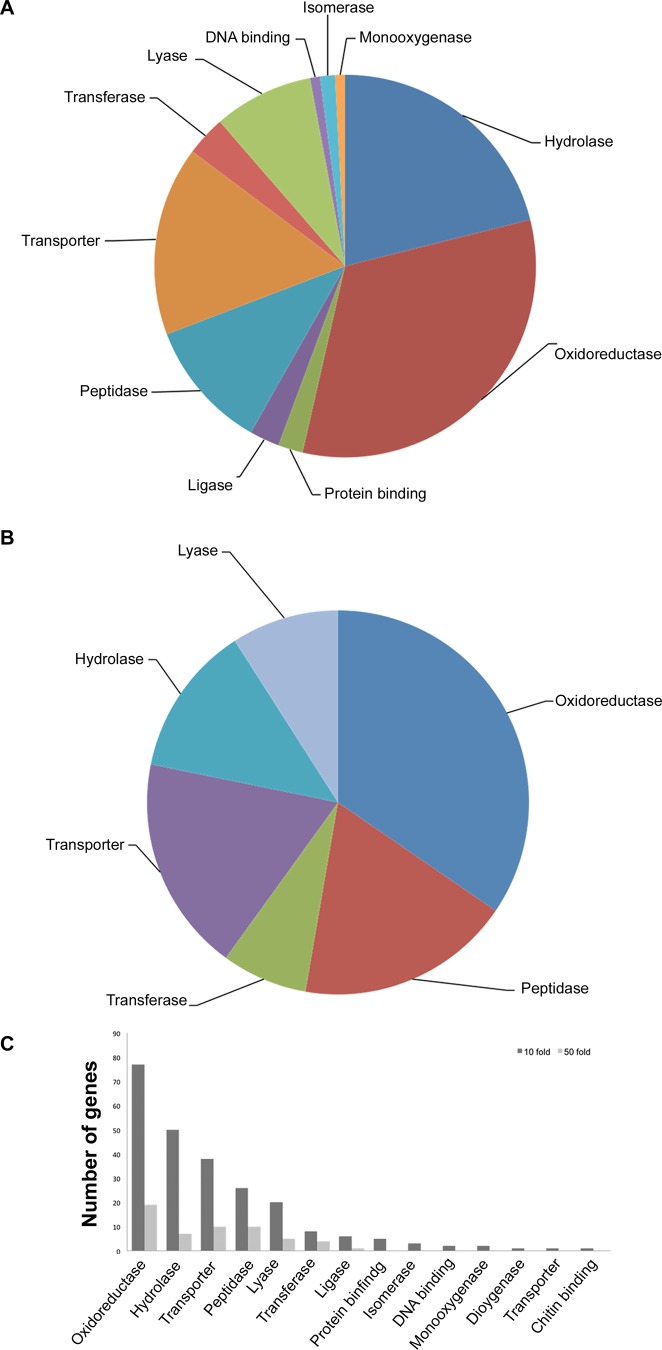
Categories of *F*. *virguliforme* genes based on BLAST2GO analyses for molecular functions. A, Categories of *F*. *virguliforme* genes with 10-fold induction. B, Categories of *F*. *virguliforme* genes with 50-fold induction. C, Percentage of genes distributed under different functional categories for molecular functions are presented as bar diagram for *F*. *virguliforme* genes with 10 and 50 fold induction over mycelia and germinating conidia.

GO molecular function term enrichment analysis was also conducted for the 369 *F*. *virguliforme* genes with ≥10-fold up-regulation. This led to the classification of 273 genes into 11 categories. The largest GO category includes genes encoding enzymes with oxidoreductase and hydrolase activities (Fig 6A and 6C). On the other hand, GO annotation for molecular functions of *F*. *virguliforme* genes with ≥50-fold induction in infected tissues revealed five classes of genes, with most genes encoding enzymes with oxidoreductase, peptidases and hydrolase activities, and transporters (Fig 6B and 6C).

### Genes with unknown function induced during infection

Ninety-six *F*. *virguliforme* genes with ≥10-fold increased expression could not be assigned to a functional category by Blast2GO analysis (S3 Table). Of these 96 genes, 64 were annotated by conducting BLASTX search. One of the genes (*g9838*), showed high identity to a stress responsive a/b barrel domain containing protein identified in *Aspergillus niger* (XP_003188860). Three polyketide synthetic genes including: snoal-like polyketide cyclase protein (*g13126*), snoal-like polyketide cyclase family protein (*g14688*) and lovastatin-like diketide synthase gene (*g14617*) were identified. Polyketides such as the T-toxin produced by *Cochliobolus heterostrophus* [33, 34] have been shown to be virulence factors and host specific toxins.

We also identified four genes (*g12373*, *g12372*, *g14510*, and *g5504*) encoding CFEM domain containing proteins. CFEM domains are unique to fungi and are found in large numbers in pathogenic ascomycetes relative to non-pathogenic ascomycetes [35]. We failed to find homologous functionally characterized genes for 32 *F*. *virguliforme* genes that showed ≥10-fold induction during infection. These genes could encode species-specific factors, some of which could be involved in virulence.

### Identification of candidate secretory proteins

The secretory peptides including virulence factors and digestive enzymes are used by fungi to attack plants. Therefore, secreted proteins are considered as candidate virulence factors involved in suppression of defense mechanisms (e.g. effector proteins), degradation of host proteins (e.g. peptidases), degradation of antifungal compounds (e.g. glyceollin detoxification enzymes) or degradation of host tissues (e.g. hydrolytic enzymes). Seventy-three of the 369 *F*. *virguliforme* genes with ≥10-fold induction in infected roots were shown to encode putative secretory proteins (S4 Table). Blast2Go analysis of these proteins for cellular components classified these genes mainly into extracellular and membrane-associated proteins. We investigated the early and late infection-specific genes encoding candidate secretory peptides/proteins (Table 3). Secretory genes induced during early infection stage (S1 Fig) encode proteins that are unlikely have hydrolytic activities; whereas, in the late infection stage many induced genes encode enzymes that have hydrolytic activities (Table 3).

**Table 3:** Differentially induced *F*. *virguliforme* genes that are predicted to encode secretory proteins during early and late infection stages.

**Gene name**	**Sequence description**	**Fold Change**			** **
		^**1**^**Early**	^**1**^**Late**	^2^**Early/Late**	^**3**^**R/C values**
g11354	Hypothetical protein AFLA_059330	82.7	7.7	10.7	2
g13527	Extracellular matrix protein precursor	5.2	1	5.2	1
g10823	Unknown	115.3	33.8	3.4	2
g9652	Triacylglycerol lipase	16.7	5.5	3	1
g35	Unknown	24.2	8.3	2.9	1
g13651	Acetolactate synthas	5	1.8	2.9	3
**Gene name**	**Sequence description**	**Fold Change**			^**3**^**R/C values**
		^**1**^**Early**	^**1**^**Late**	^2^**Late/Early**	
g4306	Pectate lyase c	0.9	33.2	38.1	1
g14206	Glycoside hydrolase family 43 protein	0.8	12.7	15.1	1
g13672	Cell wall glycosyl hydrolase	0.9	11.6	12.5	1
g13332	Lipolytic protein g-d-s-l family	1	12.4	12.4	3
g10242	Endoglucanase iv precursor	0.9	10.7	12.1	2
g10829	Expansin-like protein	1	11.7	11.7	1
g14123	Pectate lyase	1	11.1	11.1	1
g11732	Glycoside hydrolase family 10 protein	0.9	6.3	6.9	1
g12142	Glycoside hydrolase family 5 protein	2.1	11.6	5.4	4
g2034	Hypothetical protein NECHADRAFT_99942	1	5.4	5.4	5
g10874	Sugar transporter stl1	1.7	7.3	4.4	4
g3939	Para-nitrobenzyl esterase	1	3.5	3.5	1
g13138	Glycoside hydrolase family 115 protein	1.3	3.9	3	1
g12597	Cytochrome p450 monooxygenase	1	2.9	2.9	2
g225	Xylosidase: arabinofuranosidase	2.4	6.5	2.8	1
g3691	Nitrate reductase	2.3	6	2.6	2
g218	Exopolygalacturonase	2.1	4.4	2.1	1
g14347	Saponin hydrolase precursor	2.4	4.8	2	1
g10894	Hexose carrier protein	1.7	3.4	2	1
g228	Hypothetical protein FOXB_14459	1.7	3.4	2	1

^1^Fold changes in expression levels during early and late stages are calculated against the mean RPKM values of individual genes in germinating conidia and mycelia.

^2^Minimum of two-fold differences in expression levels between early and late infection stages are presented. Rest of the genes encoding putative secretory peptides or proteins can be found in S4 Table.

^3^RC values are confidence scores for secretory protein prediction. RC value 1 indicates highest confidence; while 5 the lowest.

### Identification of candidate virulence genes

Virulence genes are involved in disease development, but are not essential for completing the pathogen's life cycle [36]. In order to identify virulence genes, the sequences of the 369 genes with ≥10-fold induction during root infection were investigated to determine if any of them show high identity to any genes with known virulence functions. Twenty-seven candidate virulence genes were identified from this search (S5 Table).

Genes expressed during infection only may also be candidate virulence genes. Two-hundred and twenty-four *F*. *virguliforme* genes were expressed only in the infected soybean roots. Some of these genes showed very low RPKM values. However, 83 of the 224 genes showed high transcript levels (more than 20 reads in infected tissues) and could be considered candidate virulence genes. Blast2GO analysis for possible molecular functions of these genes showed that a majority of the 83 induced genes encode the pectate lyases and glucoside hydrolyases involved in cell wall degradation (S6 Table).

## Discussion

To better understand how *F*. *virguliforme* causes root necrosis in soybean, we conducted a comparative transcriptomic analysis to identify genes that are induced during infection of soybean roots. *F*. *virguliforme* is considered to be a hemi-biotrophic fungal pathogen with an initial biotrophic phase [8–10, 37]. To provide molecular evidence supporting this hypothesis, we developed a model system with 7-day old etiolated seedlings (Fig 1; S1 Fig; [11]). This system provides a uniform infection of root tissue by the pathogen. We observed that over 10,000 (72%) *F*. *virguliforme* genes are induced during infection as opposed to only few hundreds in our initial study of the infected roots of light-grown soybean seedlings (B.B. Sahu and M.K. Bhattacharyya, unpublished). Less than 4% of the transcripts from infected roots of etiolated soybean seedlings are *F*. *virguliforme*-specific (Fig 2). Our deep sequencing approach identified 10,626 (72%) of the 14,845 predicted *F*. *virguliforme* genes that were expressed during infection. Of these genes, 224 were expressed only in infected roots based on their absence in germinating conidia and mycelia. In addition to classifying the *F*. *virguliforme* genes based on their identities with annotated genes, we identified genes encoding putative secretory peptides/protein that may play a role in virulence. We identified 27 candidate virulence genes based on their homologies to other functionally characterized virulence genes (S5 Table).

### Genes induced during early infection stage

Over all, relatively few of the infection-inducible genes showed higher expression levels in the early infection stage as compared to that in the late infection stage. These genes may be important in establishment of the infection process. Of these genes, *g9652* (Table 3) showed high identity to a triacylglycerol lipase gene, a virulence factor gene in *Mycobacteria tuberculosis* [38, 39]. In pathogenic fungi, it has been suggested that lipases in general are involved in the penetration of the waxy cuticle [40]. It has been shown that a lipase (FGL1) determines the virulence of the wheat pathogen, *F*. *gramminerum* [41].

*F*. *virguliforme* g13527 protein (Table 3) shows high identity to an extracellular matrix protein with GPI anchored domain and a transmembrane domain found in epidermal growth factors. Extracellular matrix proteins may be involved in cell adhesion and are secreted by fungi. GPIs are membrane or cell wall proteins. GPI7 in *F*. *graminearium* governs the virulence function [42].

Phytopathogenic fungi relay signals upon sensing the cuticle. *CHIP2* and *CHIP3* encoding hard surface inducible proteins are induced in *Colletotrichum gloeosporioide* following contact with the cuticle [43]. Expression of the *F*. *virguliforme* homologue *(g12834)* of *CHIP2* during early stage infection may suggest its role in surface sensing and signaling for tissue entry (S3 Table).

Fungal pathogens use secreted extracellular enzymes to degrade structural barriers to penetrate host cells [44, 45]. Upon successful entry into the host plant, the fungi must resist plant produced antimicrobial compounds [46]. Fungi employ enzymes with oxidative-reductive properties to degrade antimicrobial compounds such as phytoalexins. In response to the pathogenic fungal attack, plants secrete polyamines [47] and phytoalexins [48] to defend against invading pathogens. Consistent with this, *F*. *virguliforme* genes encoding enzymes involved in detoxification of plant defense molecules show high expression during the early infection stages with declining transcript levels as infection progresses. Glyceollin is a soybean phytoalexin produced in response to microbial invaders [49]. The expression of the *Fusarium oxysporum* f. sp. *pisi* pisatin demethylase has been documented to detoxify pisatin, a pea phytoalexin, for establishing compatible interaction [50]. The *F*. *virguliforme g13127* gene showing high identity to pisatin demethylase was induced to a high level during infection (S5 Table). Most likely *g13127* encodes glyceollin demethylase and is involved in glyceollin metabolism. Along with phytoalexins, plants produce aromatic defense compounds that are toxic to fungi. The *F*. *virguliforme g8644* gene encodes dienelactone hydrolase (S5 Table). The enzyme is involved in the *β*-ketoadipate pathway that catabolizes aromatic compounds into acetyl co-A and succinyl co-A [51–53].

### Expression of toxin genes for foliar SDS development

As the fungus switches to the necrotrophic phase it produces toxins and necrosis inducing factors and starts killing plant cells for rapid nutrient uptake and growth. Trichothecene is a mycotoxin that severely impacts protein synthesis by inhibiting either the initiation or the elongation process of translation by interfering with peptidyl transferase activity [54]. Hydroxylation at carbon-2 (C-2) is the first committed intermediate of the trichodiene pathway and is mediated by the cytochrome P450 monooxygenase [55]. Expression of multiple cytochrome p450 related genes (*g5125*, *g9837*, *g9839*, *g9830*) increased in this pathogen during the different stages of infection (S1 and S5 Tables). In addition to the role in the trichodiene pathway, cytochrome P450 enzymes are considered to be involved in fungal adaptations [56, 57]. The related cytochrome P450 genes are arranged in a group in the *F*. *virguliforme* genome. This grouped arrangement suggests that these genes may be coordinately expressed and operate as a cassette.

To date at least 12 potential toxins have been identified in *F*. *virguliforme* including proteinacious FvTox1, non-ribosomally produced peptides, polyketides and effector proteins [11, 12, 14]. We observed increased expression of necrosis inducing peptide 2 (*FvNIP2*, *g12151)* during infection of *F*. *virguliforme*, thus identifying it as a potential *F*. *virguliforme* toxin (S1 and S5 Tables; [14]). We did not find increased expression of *FvTox1* (*g6924*) in infected root. FvTox1 has been shown to be important for foliar SDS symptom development [11, 17]. This gene is however constitutively expressed in the fugal mycelia and germinating spores (S1 Table).

### Induction of genes for root necrosis during late infection phase

The necrotrophic phase in infected soybean roots presumably begins with the accumulation of hydrolytic and glycosyl hydrolases to cause root necrosis. In our model system, roots of etiolated seedlings showed very little visible signs of root necrosis at early stage of infection; however, in the late infection stage root rotting due to necrosis was observed (S1 Fig). Among the genes encoding candidate virulence peptides/proteins with secretory signals (S3 Table), only a few are strongly induced in the early infection stage; whereas, a large number of genes encoding secretory proteins are induced during late infection stage (Table 3). The pattern of gene expression completely changed as *F*. *virguliforme* entered the necrotrophic phase. A large number of pectate lyases with secretary signals involved in degradation of the cell wall component pectin are induced (Table 3). Among the infection-induced genes, a majority showed increasing levels of expression during the late stage of infection. The genes with enhanced expression during late stage infection include functional categories such as localization, cell hydrolysis, oxidation-reduction, membrane transport, many degradative enzymes such as pectate lyases, glucoside hydrolases, and elastinolytic metalloproteinase (Figs 5A, 6A and 6B; S4 and S6 Tables).

Investigation of the infection-induced (i) genes encoding putative secretory peptides/proteins (Table 3; S4 Table), (ii) genes specifically induced during infection (S6 Table) or (iii) infection-induced genes with homology to functionally characterized virulence genes (S5 Table), strongly suggest that during the late infection stage a large number of genes encoding cell wall degrading enzymes are induced. Expression of genes encoding degradative enzymes is common in other necrotrophic fungi. Necrotrophic fungi have also been shown to use a wide array of cell wall degrading enzymes to breakdown host tissues for penetration. Taken together, this suggests that degradative enzymes may play a major role in changing the fungus from its biotrophic phase to the necrotrophic phase. In the case of the pea pathogen *Nectria haematococca*, pathogenesis associated protein PEP2 has been shown to play a role in infection [58]. PEP2 is possibly involved in degrading the extracellular matrix proteins, and cleavage of the cell surface receptors. The *F*. *virguliforme* gene *g8640*, with high identity to *PEP2* and induced during infection of soybean roots, may be involved in degradation of soybean root tissues (Fig 6; S1 and S5 Tables).

Plants cell walls are composed of complex carbohydrate and degradation of these complex walls requires specialized carbohydrate degrading enzymes. Hemicellulose constitutes a large fraction of the cell wall and contains xyloglucan, glucuronoarabinoxylan, mannan, galactan, arabinan, mixed-linked glucan, and glucuronoarabinoxylan [59]. During infection, necrotrophic pathogens degrade these building blocks by increasing the secretion of cell wall degrading enzymes such as pectate lyase and polygalacturonases [60–62].

Many cell wall degrading enzymes were found to be induced during infection with a dramatic increase in expression during the late infection phase, presumably to degrade the plant cells when the fungus becomes necrotrophic. In *F*. *virguliforme*, two pectate lyase genes (*g7676* and *g11622*), one exopolygalacturonase gene (*g4533*), one endo-beta-xylanase (*g11621*), and one cellobiohydrolase ii (*g14515*) gene were induced during late infection stage (S1, S4 and S6 Tables). Endo-β-1,4-xylanase has been shown to be important for infection by *Fusarium oxysporum* f. sp. *lycopersici* [63].

*F*. *virguliforme* gene *g14032* encoding a putative elastinolytic metalloproteinase Mep is highly induced during late infection (S5 Table). Mep proteins belong to a family of proteins likely involved in degradation of plant tissues [64]. Increased accumulation of *g14032* transcripts during late stage infection suggest that it may be involved in transitioning the pathogen from its biotrophic to the necrotrophic phase.

As the carbohydrates of the cell walls are degraded, the fungi encounter cell wall associated peptides that could be toxic. Following infection of soybean, *F*. *virguliforme*, genes *g8474*, and *g10809* encoding carboxypeptidases, and *g12211* and *g12135* encoding leucyl aminopeptidases were induced (S1 and S5 Tables). Carboxypeptidases are specific proteases which hydrolyze the peptide bond of an amino acid at the C-terminus [65], whereas the leucyl aminopeptidases catalyze the hydrolysis of the leucine residues at the N-terminus. These infection-induced *F*. *virguliforme* genes may be involved in digesting the cell-wall associated peptides.

After successful entry the fungal pathogens travel through the intra and intercellular passages and come in contact with the structural proteins and enzymes important for host defense. Enzymes such as subtilases and alkaline proteinases have been shown to degrade defensive enzymes [66]. Increased expression of *F*. *virguliforme* gene *g14667* encoding subtilase in the infected tissues especially during late infection suggests its possible involvement in nullifying host defense-related enzymes (S5 Table).

## Conclusions

In this comparative transcriptomic study, we were able to identify putative virulence factors by the following approaches: (i) investigation of the functions of infection-induced genes based on sequence homology using Blast2GO analyses; (ii) identification of secretory proteins and their GO annotation for possible functions; (iii) search for candidate virulence genes through sequence homology search with functionally characterized virulence genes; and (iv) studying the genes that are only induced in infected roots. Expression of several infection-induced genes encoding enzymes with oxidation-reduction properties for degradation of antimicrobial compounds such as the phytoalexin glyceollin could be an important virulence mechanism in this pathogen during early biotrophic phase. Induced expression hydrolytic and cell wall degrading enzyme genes (pectate lyase, glycoside hydrolase, polygalacturonases) in *F*. *virguliforme* during soybean root infection parallels the similar observations made recently in the transition of multiple *Zymoseptoria tritici* genotypes [67]. Expression of a large number of genes encoding enzymes with catalytic and hydrolytic activities during late infection stage suggests that cell wall degradation is involved in establishing the necrotrophic phase in this pathogen. This study suggests that enzymes with hydrolytic and catalytic activities play an important role in the transitioning the pathogen from biotrophic to necrotrophic phase.

## Supporting Information

S1 Fig Responses of roots of etiolated soybean seedlings to *F*. *virguliforme* Mont-1 infection. (TIF)

S2 Fig Relative expression of *FvTox1* following *F*. *virguliforme* infection. Relative expression of *FvTox1* was determined by conducting qRT-PCR at 1-d, 3-d, 5-d and 10-d post inoculation with *F*. *virguliforme* Mont-1 conidia. Relative gene expression levels was compared with the expression level at 1-d post *F*. *virguliforme* inoculation. Constitutively expressed *F*. *virguliforme GAPDH* (g2019) (S2 Table) was used for normalization of the FvTox1 expression levels. Data are means and standard deviations (SD) of two independent biological replications with three technical replications (n = 6).
(TIF)

S1 Table RPKM values of *F*. *virguliforme* transcripts induced during infection of soybean roots. (XLSX)

S2 Table Primers used in RT-PCR and qRT-PCR analyses. (XLSX)

S3 Table Annotation of *F*. *virguliforme* genes that failed to show homologies to previously annotated genes used in Blast2GO analyses. (XLSX)

S4 Table *F*. *virguliforme* proteins with secretory signals. (XLSX)

S5 Table Infection-induced *F*. *virguliforme* genes showing high identity to known virulence genes. (DOCX)

S6 Table Infection specific genes with hydrolase activities. (XLSX)
