# Supplementary figures and images for: Investigation of the Fusarium virguliforme Transcriptomes Induced during Infection of Soybean Roots Suggests that Enzymes with Hydrolytic Activities Could Play a Major Role in Root Necrosis
Source: PLoS One. 2017 Jan 17;12(1):e0169963. doi: 10.1371/journal.pone.0169963 (PMC5241000; doi:10.1371/journal.pone.0169963)

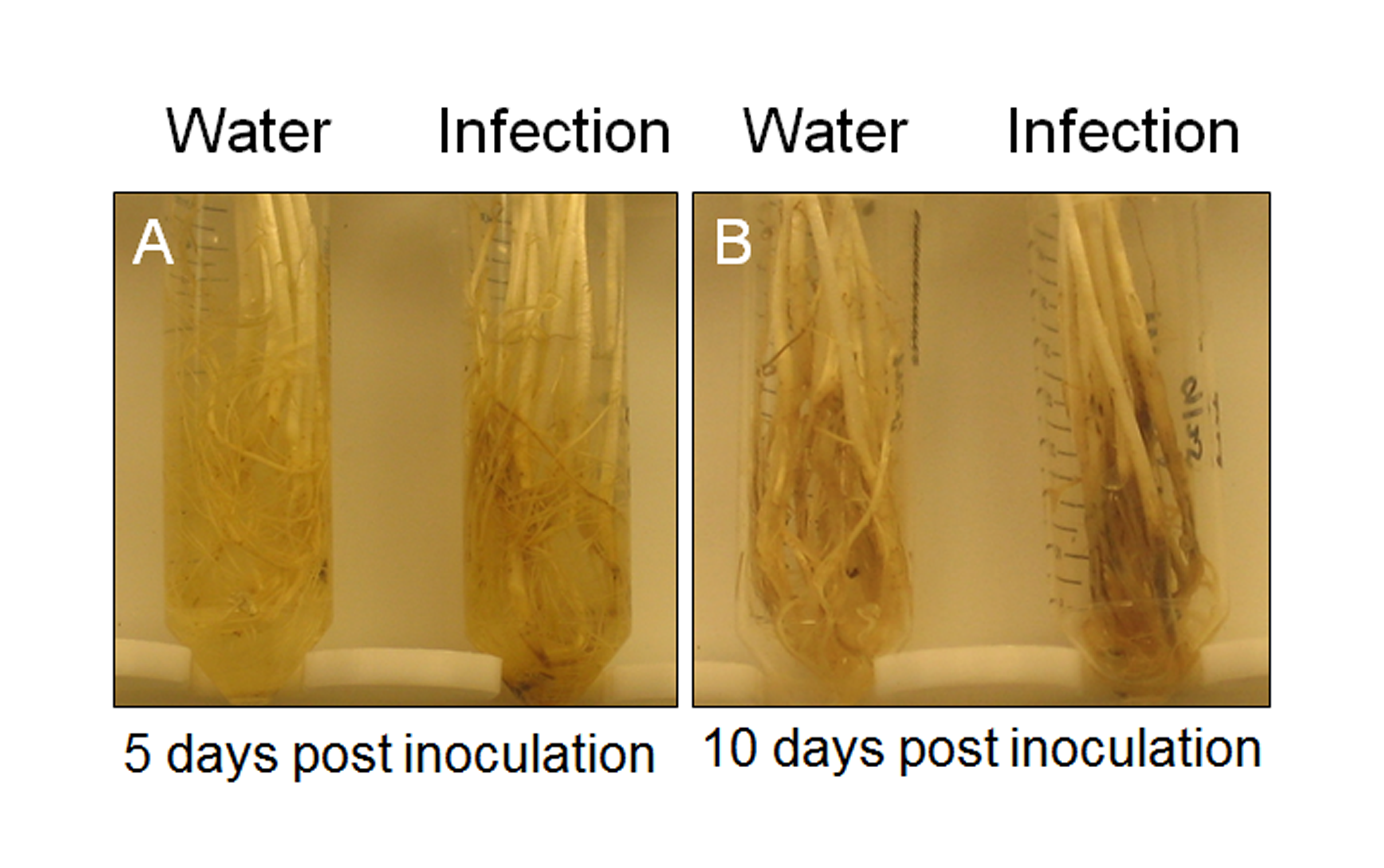

Supplement: S1 Fig — (TIF) [file pone.0169963.s001.tif]

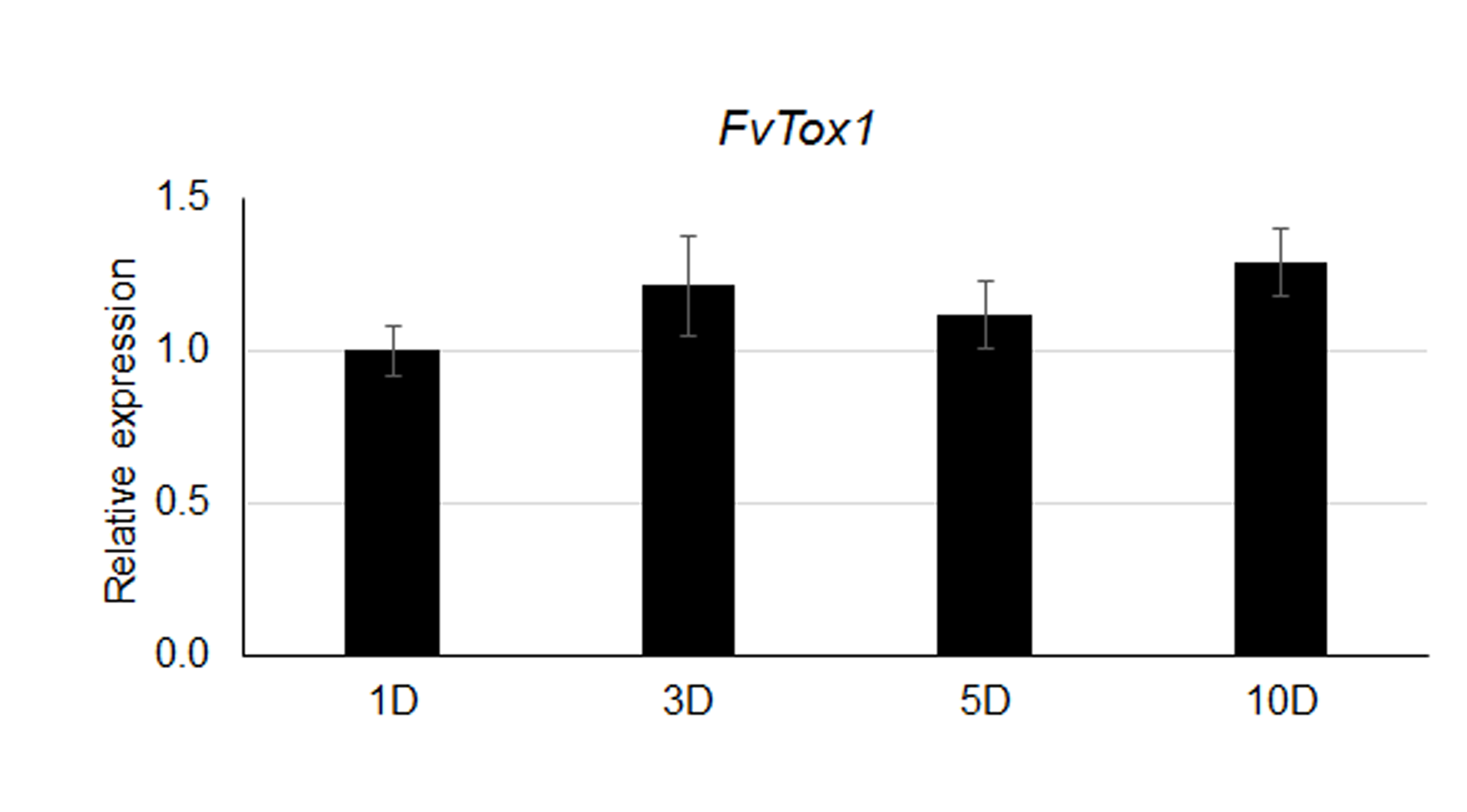

Supplement: S2 Fig — Relative expression of FvTox1 was determined by conducting qRT-PCR at 1-d, 3-d, 5-d and 10-d post inoculation with F. virguliforme Mont-1 conidia. Relative gene expression levels was compared with the expression level at 1-d post F. virguliforme inoculation. Constitutively expressed F. virguliforme GAPDH (g2019) (S2 Table) was used for normalization of the FvTox1 expression levels. Data are means and standard deviations (SD) of two independent biological replications with three technical replications (n = 6). (TIF) [file pone.0169963.s002.tif]
