# Supplementary material for: Investigation of the Fusarium virguliforme Transcriptomes Induced during Infection of Soybean Roots Suggests that Enzymes with Hydrolytic Activities Could Play a Major Role in Root Necrosis
Source: PLoS One. 2017 Jan 17;12(1):e0169963. doi: 10.1371/journal.pone.0169963 (PMC5241000; doi:10.1371/journal.pone.0169963)
Supplement: S5 Table — (DOCX) [file pone.0169963.s007.docx]

**S5 Table. Infection-induced *F. virguliforme* genes showing high identity to known virulence genes.**

| **Sl. No.** | **^1^Gene ID** | **Name of the gene** | **Reference (s)** |
| --- | --- | --- | --- |
| **^2^Establishment of localization and hydrolysis of cell wall building block** | | | |
| 1 | g8640 | Pathogenesis associated protein pep2 | [[58](#_ENREF_58)] |
| 2 | ^3^g14032 | Elastinolytic metalloproteinase | [[64](#_ENREF_64)] |
| 3 | ^3^g7676 | Pectate lyase, and polygalacturonases | [[60-62](#_ENREF_60)] |
| 4 | g14667 | Subtilases and alkaline proteinases | [[66](#_ENREF_66)] |
| 5 | ^3^g13886 | 3-(3-hydroxy-phenyl) propionate hydroxylase | [[68](#_ENREF_68)] |
| 6 | ^3^g12501 | Acetyl xylan esterase and endo-beta-xylanase | [[69](#_ENREF_69), [70](#_ENREF_70)] |
| 7 | ^3^g14546 | Cellulose-binding family ii | [[71](#_ENREF_71)] |
| 8 | ^3^g14515 | Cellobiohydrolase ii | [[72](#_ENREF_72)] |
| 9 | g11698 | Glycosyl hydrolase | [[73](#_ENREF_73)] |
| 10 | g8474 | Carboxypeptidase | [[65](#_ENREF_65)] |
| **Oxidation-reduction process** | | | |
| 11 | ^3^g13127 | Pisatin demethylase | [[50](#_ENREF_50)] |
| 12 | ^3^g8644 | Dienelactone hydrolase | [[51-53](#_ENREF_51)] |
| 13 | g5125 | Cytochrome P450 monooxygenase | [[55](#_ENREF_55)] |
| 14 | ^3^g12373 | CFEM domain containing proteins | [[74](#_ENREF_74)] |
| 15 | ^3^g11119 | Aldehyde dehydrogenase | [[75](#_ENREF_75)] |
| 16 | g13120 | Catechol dioxygenases | [[76](#_ENREF_76)] |
| **Transmembrane transport** | | | |
| 17 | g12034 | Aminoacid permease, arginine permease | [[77](#_ENREF_77)] |
| 18 | g9707 | *β*-galactosidase | [[78](#_ENREF_78)] |
| **Primary metabolic process** | | | |
| 19 | g12847 | Acetylornithine aminotransferase | [[79](#_ENREF_79)] |
| 20 | g3575 | Acyl-dehydrogenase | [[80](#_ENREF_80)] |
| **Catalytic activity** | | | |
| 21 | ^3^g2961 | Chitin synthase | [[81](#_ENREF_81)] |
| 22 | ^3^g11621 | Endo-beta-xylanase, extracellular elastinolytic metalloproteinase, endoglucanase iv precursor, pectate lyase b and metalloprotease | [[82](#_ENREF_82), [83](#_ENREF_83)] |
| **Ion binding** | | | |
| 23 | g10929 | Indoleamine -dioxygenase family protein | [[84](#_ENREF_84)] |
| ^4^**Genes with unknown functions** | | | |
| 24 | ^3^g10823 | Nuclear transcription x-box binding-like 1 | [[85](#_ENREF_85)] |
| 25 | ^3^g9838 | Stress responsive a/b barrel domain-containing proteins | [[86](#_ENREF_86)] |
| 26 | g12151 | Necrosis and ethylene inducing peptide 1 precursor | [[14](#_ENREF_14)] |
| 27 | ^3^g14723 | Lignostilbene dioxygenase | [[87](#_ENREF_87), [88](#_ENREF_88)] |
| ^1^ The Gene IDs are as in Srivastava et al. 2014.  ^2^The genes are presented by their functions.  ^3^Genes expressed in infected root tissues, but not in germinating conidia and/or mycelia.  ^4^ Genes with unknown functions were annotated at NCBI using the BlastX program.  **References**  68. Ferrández A, Garciá JL, Díaz E. Genetic characterization and expression in heterologous hosts of the 3-(3-hydroxyphenyl)propionate catabolic pathway of *Escherichia coli* K-12. J Bacteriol. 1997; 179: 2573-2581.  69. Biely P, Puls J, Schneider H. Acetyl xylan esterases in fungal cellulolytic systems. FEBS Lett. 1985; 186: 80-84.  70. Gómez-Gómez E, Ruı́z-Roldán MC, Di Pietro A, Roncero MIG, Hera C. Role in pathogenesis of two endo-β-1,4-xylanase genes from the vascular wilt fungus *Fusarium oxysporum*. Fungal Genet Biol. 2002; 35: 213-222.  71. Tomme P, Van Tilbeurgh H, Pettersson G, Van Damme J, Vandekerckhove J, Knowles J, et al. Studies of the cellulolytic system of *Trichoderma reesei* QM 9414. Euro J Biochem. 1988; 170: 575-581. doi: 10.1111/j.1432-1033.1988.tb13736.x.  72. Barr BK, Hsieh Y-L, Ganem B, Wilson DB. Identification of two functionally different classes of exocellulases. Biochem. 1996; 35: 586-592. doi: 10.1021/bi9520388.  73. Faure D. The family-3 glycoside hydrolases: from housekeeping functions to host-microbe interactions. Appl Environ Microbiol. 2002; 68: 1485-1490. doi: 10.1128/aem.68.4.1485-1490.2002.  74. Kulkarni RD, Kelkar HS, Dean RA. An eight-cysteine-containing CFEM domain unique to a group of fungal membrane proteins. Trends Biochem Sci. 2003; 28: 118-121.  75. Prado-Cabrero A, Schaub P, Díaz-Sánchez V, Estrada AF, Al-Babili S, Avalos J. Deviation of the neurosporaxanthin pathway towards β-carotene biosynthesis in *Fusarium fujikuroi* by a point mutation in the phytoene desaturase gene. FEBS J. 2009; 276: 4582-4597. doi: 10.1111/j.1742-4658.2009.07164.x.  76. Harayama S, Kok M, Neidle EL. Functional and evolutionary relationships among diverse oxygenases. Annu Rev Microbiol. 1992; 46: 565-601. doi: doi:10.1146/annurev.mi.46.100192.003025.  77. Trip H, Evers ME, Driessen AJM. PcMtr, an aromatic and neutral aliphatic amino acid permease of *Penicillium chrysogenum*. BBA-Biomembranes. 2004; 1667: 167-173.  78. Macris BJ, Markakis P. Characterization of extracellular β-d-galactosidase from *Fusarium moniliforme* grown in whey. Appl Environ Microbiol. 1981; 41: 956-958.  79. Ledwidge R, Blanchard JS. The dual biosynthetic capability of N-acetylornithine aminotransferase in arginine and lysine biosynthesis. Biochemistry. 1999; 38: 3019-3024. doi: 10.1021/bi982574a.  80. Maggio-Hall LA, Lyne P, Wolff JA, Keller NP. A single acyl-CoA dehydrogenase is required for catabolism of isoleucine, valine and short-chain fatty acids in *Aspergillus nidulans*. Fungal Genet Biol. 2008; 45: 180-189.  81. Martín-Udíroz M, Madrid MP, Roncero MIG. Role of chitin synthase genes in *Fusarium oxysporum*. Microbiology. 2004; 150: 3175-3187. doi: 10.1099/mic.0.27236-0.  82. West CA. Fungal elicitors of the phytoalexin response in higher plants. Naturwissenschaften. 1981; 68: 447-457. doi: 10.1007/bf01047514.  83. Rathjen JP, Chang JH, Staskawicz BJ, Michelmore RW. Constitutively active Pto induces a Prf-dependent hypersensitive response in the absence of avrPto. EMBO J. 1999; 18: 3232-3240.  84. Cho Y, Ohm RA, Grigoriev IV, Srivastava A. Fungal-specific transcription factor AbPf2 activates pathogenicity in *Alternaria brassicicola*. Plant J. 2013; 75: 498-514.  85. Gupta SK RA, Kanwar SS, Sharma TR. Comparative analysis of zinc finger proteins involved in plant disease resistance. PLoS ONE 2012; 7: e42578.  86. Hegge A, Lønborg R, Nielsen DM, Sørensen JL. Factors influencing production of Fusaristatin A in *Fusarium graminearum*. Metabolites. 2015; 5: 184-191.  87. Greene GH, McGary KL, Rokas A, Slot JC. Ecology drives the distribution of specialized tyrosine metabolism modules in fungi. Genome Biology and Evolution. 2014; 6: 121-132. doi: 10.1093/gbe/evt208. PubMed PMID: PMC3914699.  88. Potnis N, Krasileva K, Chow V, Almeida NF, Patil PB, Ryan RP, et al. Comparative genomics reveals diversity among xanthomonads infecting tomato and pepper. BMC Genomics. 2011; 12: 1-23. doi: 10.1186/1471-2164-12-146. | | | |
